# Cardiovascular Safety Signals of Osteoporosis Therapies: A FAERS Pharmacovigilance Study

**DOI:** 10.31138/mjr.250126.psj

**Published:** 2026-06-16

**Authors:** Manush Sondhi, Namrata Singh, Julie Carkin, Grant Hughes

**Affiliations:** Division of Rheumatology, Department of Medicine, University of Washington School of Medicine, Seattle, WA, United States

**Keywords:** osteoporosis, cardiovascular diseases, bisphosphonates, romosozumab, zoledronic acid

## Abstract

**Objective:**

To evaluate the associations between commonly used osteoporosis medications and major adverse cardiovascular events (MACE) using data from the Food and Drug Administration (FDA) Adverse Event Reporting System (FAERS).

**Methods:**

We conducted a retrospective pharmacovigilance analysis of FAERS reports between January 2019 and December 2024, identifying all reports involving alendronate, risedronate, zoledronic acid, denosumab, teriparatide, abaloparatide, or romosozumab. MACE outcomes included myocardial infarction (MI), stroke, atrial fibrillation (A-fib), and cardiac failure (CF), defined using Medical Dictionary for Regulatory Activities Preferred Terms. Disproportionality was assessed using reporting odds ratios (RORs), with signals defined by a lower 95% confidence interval >1. All analyses were descriptive and hypothesis-generating.

**Results:**

FAERS contained more than 30 million adverse event reports during the study period. Romosozumab demonstrated the highest ROR for MI (2.38) and stroke (3.72), and also showed elevated signals for A-fib and CF (ROR 3.76). Zoledronic acid showed notable associations with stroke (ROR 2.42) and A-fib (ROR 3.22). Denosumab and abaloparatide were associated with the lowest RORs across all cardiovascular outcomes. Alendronate and risedronate demonstrated intermediate disproportionality signals, particularly for A-fib and CF.

**Conclusion:**

Romosozumab and zoledronic acid demonstrated prominent cardiovascular disproportionality signals in FAERS, whereas denosumab, abaloparatide, and teriparatide showed more favorable reporting profiles. These findings should not discourage osteoporosis treatment but underscore the importance of individualised cardiovascular risk assessment and the need for further prospective evaluation to inform safe therapy selection.

## INTRODUCTION

Osteoporosis is a major global public health problem and a leading cause of fragility fractures, disability, loss of independence, and mortality in aging populations.^1^ Pharmacologic treatment with antiresorptive agents such as bisphosphonates and denosumab, and anabolic therapies such as teriparatide, abaloparatide, and romosozumab, is central to reducing fracture risk and improving quality of life.^1–3^ As these agents are increasingly used in older adults with multiple comorbidities, their long-term cardiovascular safety has become an important clinical concern.

The cardiovascular effects of osteoporosis therapies have been evaluated in randomised trials, meta-analyses, and observational studies, but the results are heterogeneous and at times conflicting. A recent systematic review and Bayesian network meta-analysis of randomised trials in postmenopausal women found that romosozumab may be associated with a higher risk of major adverse cardiovascular events (MACE) compared with select other osteoporosis agents, although estimates were based on relatively few events.^4^ In the ARCH trial, romosozumab followed by alendronate reduced fracture risk more than alendronate alone but was associated with higher rates of serious cardiovascular events, including myocardial infarction and stroke.^5,6^ Denosumab, a receptor activator of nuclear factor κB ligand inhibitor, has been widely studied across multiple indications, and pooled trial data and meta-analyses have generally suggested a neutral cardiovascular profile, without a clear increase in myocardial infarction or stroke.^4,7–9^

For bisphosphonates, including alendronate and zoledronic acid, the relationships with MACE remain uncertain. A meta-analysis of randomised trials did not demonstrate a consistent increase in overall cardiovascular events with bisphosphonate therapy, but several studies noted signals for atrial fibrillation, particularly with zoledronic acid.^10,11^ In the HORIZON Pivotal Fracture Trial, once-yearly zoledronic acid treatment reduced fracture risk but was associated with a higher incidence of serious atrial fibrillation.^12^ Subsequent registry-based studies have produced variable findings, with some suggesting modestly increased atrial fibrillation risk in the first year after zoledronic acid infusion.^13,14^ Observational data evaluating cardiovascular and skeletal safety of zoledronic acid in routine practice have further illustrated the complexity of disentangling drug effects from underlying comorbidity.^15^ Parathyroid hormone analogs, such as abaloparatide and teriparatide, have not been associated with a strong or consistent cardiovascular event signal in clinical trials, although the duration of exposure and sample sizes are limited. Similarly, in terms of MACE risk, oral bisphosphonates such as alendronate and risedronate have generally been considered neutral or even potentially beneficial in some observational studies, though concerns regarding atrial fibrillation persist.^10–11,16^

Importantly, most of the available evidence comes from randomised trials and select observational cohorts. These data may not fully capture rare or delayed events, nor fully represent real-world populations with multiple comorbidities, polypharmacy, and heterogeneous cardiovascular risk. In this context, large pharmacovigilance databases such as the Food and Drug Administration Adverse Event Reporting System (FAERS) can provide complementary insights into post-marketing safety. FAERS collects millions of spontaneous reports submitted by healthcare professionals, patients, and manufacturers, and is widely used for hypothesis-generating signal detection through disproportionality analyses such as the reporting odds ratio (ROR).^17–20^

The cardiovascular risks associated with osteoporosis medications therefore remain incompletely understood, particularly in routine clinical practice.^4,10,21^ Given the high prevalence of osteoporosis among patients managed in rheumatology practice, including those with inflammatory arthritis, glucocorticoid exposure, and multimorbidity, understanding the cardiovascular safety profiles of osteoporosis therapies is of direct relevance to rheumatologists.^2,3^ Against this backdrop, we hypothesised that commonly used osteoporosis medications would demonstrate differential cardiovascular reporting patterns in real-world pharmacovigilance data. Specifically, our study aimed to evaluate the association between commonly used osteoporosis medications and MACE reporting in the FAERS database. We sought to characterise reporting patterns of myocardial infarction (MI), stroke, atrial fibrillation (A-fib), and cardiac failure (CF) associated with alendronate, risedronate, zoledronic acid, denosumab, teriparatide, abaloparatide, and romosozumab. We also aimed to evaluate whether individual agents are associated with disproportional reporting of MACE in the FAERS database and to compare these patterns across antiresorptive and anabolic therapies. By leveraging a large national pharmacovigilance database, we aim to better define the cardiovascular safety profiles of these therapies in real-world use and support individualised treatment decisions that balance fracture prevention with cardiovascular risk.

## METHODS

This was a retrospective pharmacovigilance study utilising adverse event reports from FAERS between January 2019 and December 2024. FAERS is a large, publicly accessible postmarketing surveillance database that aggregates voluntary (patients, health care professionals) and mandatory (drug manufacturer) reports of adverse events, medication errors, and product quality issues associated with drugs and biologics approved in the United States.^17^,^18^ Each report may include patient demographics, indication for therapy, suspect and concomitant drugs, and clinical outcomes; all data are anonymised and de-identified. As the database contains no identifiable patient information, institutional review board approval and informed consent were not required. Permission to use FAERS data is granted under public access policies of the U.S. Food and Drug Administration.

We identified all reports in which any of the following osteoporosis medications was listed as a suspect or interacting drug: alendronate, risedronate, zoledronic acid, denosumab, teriparatide, abaloparatide, or romosozumab. Only agents approved by the FDA in or before 2019 were included to ensure adequate postmarketing experience during the study period. Reports involving individuals younger than 18 years were excluded, as osteoporosis treatment or MACE in children is rare. The primary outcomes of interest were major adverse cardiovascular events, defined in this analysis as MI, stroke, A-fib, and CF. These events were identified using Preferred Terms from the Medical Dictionary for Regulatory Activities (MedDRA). For each outcome, we compiled a list of relevant Preferred Terms (for example, “myocardial infarction”, “cerebral infarction”, “ischemic stroke”, “atrial fibrillation”, “cardiac failure congestive”) and queried FAERS to capture all reports matching these terms. When a single case report contained more than one cardiovascular event, each event contributed to the corresponding outcome specific analysis.

To evaluate potential associations between individual osteoporosis medications and each cardiovascular outcome, we performed disproportionality analyses using the reporting odds ratio, an established pharmacovigilance signal detection method.^19,20,22^ The ROR compares the odds of reporting a specific adverse event with a given drug to the odds of reporting the same event with all other drugs in the database. For each drug-event pair, we constructed a 2×2 contingency table with the following cells: (A) number of reports with both the drug of interest and the adverse event of interest, (B) number of reports with the drug of interest and other adverse events, (C) number of reports with other drugs and the adverse event of interest, and (D) number of reports with other drugs and other adverse events. The ROR was calculated as (A×D)/(B×C).^19,20,22^ A safety signal was considered statistically significant when the lower bound of the 95 percent confidence interval (CI) for the ROR exceeded 1.0, indicating that the adverse event was reported more frequently with the drug of interest than would be expected based on the overall FAERS reporting distribution.^17,19^ Because FAERS includes a blend of reports from patients, health care professionals, and drug manufacturers, exposure denominators, time at risk, and detailed clinical covariates are not consistently available. As a result, we did not perform multivariable adjustment, and all analyses should be interpreted as descriptive and hypothesis generating only.^17,18,23^

## RESULTS

During the study period, the FAERS database contained more than 30 million adverse event reports involving at least one of the seven osteoporosis medications of interest.

We analysed the characteristics and disproportionality of major adverse cardiovascular events (MACE) reported in association with each osteoporosis medication; RORs with 95% confidence intervals are summarised in **Table 1**.

**Table 1.** Table depicting the calculated RORs of MACE associated with the medications used for the treatment of osteoporosis.

**Drug**	**Myocardial Infarction**	**Stroke**	**Atrial Fibrillation**	**Cardiac Failure**
Alendronate	1.13 (0.94–1.35)	1.56 (1.35–1.81)	2.69 (2.25–3.21)	2.51 (2.20–2.87)
Risedronate	0.74 (0.47–1.15)	1.29 (0.94–1.77)	1.55 (0.97–2.46)	1.86 (1.37–2.52)
Zoledronate	0.67 (0.54–0.84)	2.42 (2.17–2.69)	3.22 (2.76–3.74)	1.14 (0.95–1.36)
Denosumab	0.59 (0.51–0.68)	1.01 (0.91–1.11)	1.20 (1.03–1.39)	0.70 (0.61–0.81)
Teriparatide	0.69 (0.54–0.89)	1.32 (1.12–1.57)	1.69 (1.33–2.15)	0.73 (0.56–0.95)
Abaloparatide	0.37 (0.27–0.50)	0.67 (0.54–0.83)	1.95 (1.59–2.40)	0.25 (0.17–0.37)
Romosozumab	2.38 (2.05–2.76)	3.71 (3.31–4.16)	2.95 (2.41–3.61)	3.75 (3.29–4.27)

For MI, elevated RORs were observed only for romosozumab (ROR 2.38, 95% CI 2.05–2.76). For all other drugs, the ROR was < 1 or the 95% CI included 1 (**Table 1**).

For stroke, elevated RORs were observed for several drugs, with the exception of risedronate, abaloparatide, and denosumab. Positive disproportionality signals were observed for romosozumab (ROR 3.71, 95% CI 3.31–4.16) and zoledronic acid (ROR 2.42, 95% CI 2.17–2.69). Abaloparatide demonstrated an ROR below unity (ROR 0.67, 95% CI 0.54–0.83) (**Table 1**). When atrial fibrillation was examined, positive disproportionality signals were observed for all drugs except risedronate. Zoledronic acid demonstrated a positive disproportionality signal (ROR 3.22, 95% CI 2.76–3.74), consistent with prior reports of atrial fibrillation associated with intravenous bisphosphonate therapy.^11–15^ RORs for romosozumab, alendronate, and denosumab were elevated to a lesser degree (**Table 1**).

For CF, only three drugs showed elevated RORs. Similar to stroke (above), romosozumab exhibited the highest elevation in ROR (3.75, 95% CI 3.29–4.27), followed by alendronate (2.51, 95% CI 2.20–2.87) and risedronate (1.86, 95% CI 1.37–2.52) (**Table 1**).

**Figure 1.**
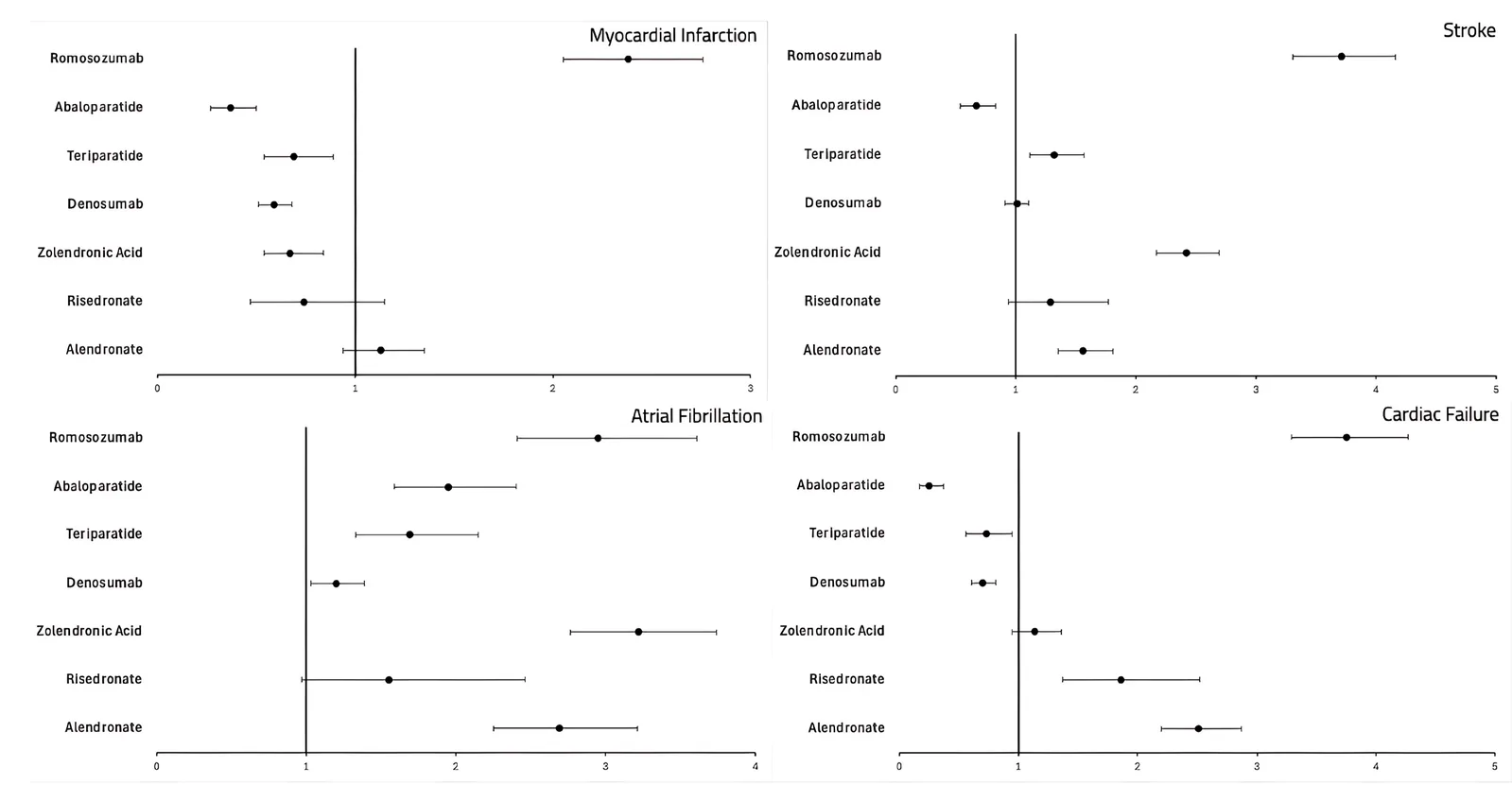
Disproportionality signals for all four MACE outcomes.

Of all drugs analysed, only romosozumab demonstrated positive disproportionality signals for all four MACE outcomes (**Figure 1**). In contrast, denosumab and abaloparatide demonstrated positive disproportionality signals for only one outcome (A-fib). Among bisphosphonates, cardiovascular reporting patterns were heterogeneous. Zoledronic acid demonstrated positive disproportionality signals for atrial fibrillation and stroke, whereas alendronate and risedronate demonstrated signals for fewer cardiovascular outcomes, most notably atrial fibrillation and cardiac failure, rather than a uniform class effect (**Figure 1**).

## DISCUSSION

In this FAERS-based pharmacovigilance analysis, we identified MACE safety signals for seven commonly used osteoporosis therapies. Some of these drug– event pairings have been reported previously, while others may be new. Overall, our findings highlight differences in cardiovascular reporting patterns across osteoporosis medications in routine clinical use and underscore the value of pharmacovigilance data in complementing evidence from randomised trials and observational studies.

Romosozumab demonstrated consistently elevated RORs across all four MACE outcomes evaluated. The presence of disproportionality signals spanning MI, stroke, A-fib, and CF suggests a pattern of increased cardiovascular event reporting relative to overall FAERS reporting distributions. From a mechanistic perspective, romosozumab inhibits sclerostin, a regulator of the Wnt signaling pathway involved in bone formation.^6^ Experimental and genetic studies suggest that sclerostin may also play roles in vascular biology and vascular calcification, raising the possibility that modulation of this pathway could influence cardiovascular risk, although causal relationships remain uncertain.^21^ Results from future studies using longitudinal data with adjudicated outcomes and robust confounder adjustment will be essential to clarify these associations and inform the safe use of romosozumab, particularly among patients with elevated baseline MACE risk.^4–6^ Across bisphosphonates, cardiovascular reporting patterns were heterogeneous rather than reflective of a uniform class effect. Zoledronic acid, an IV bisphosphonate, demonstrated positive disproportionality signals for A-fib and stroke in the FAERS database. Prior observational and pharmacovigilance studies have reported an association between IV bisphosphonates and A-fib, including analyses from VigiBase and large propensity-matched cohorts comparing zoledronic acid with denosumab, which demonstrated a modestly higher incidence of A-fib with zoledronic acid but no corresponding increase in stroke or TIA risk.^11–15^

In contrast, oral bisphosphonates demonstrated more limited and variable cardiovascular reporting patterns. Alendronate and risedronate showed positive disproportionality signals primarily for A-fib and CF, without consistent signals across all MACE outcomes.^10,11,16^

In contrast, denosumab and abaloparatide showed elevated RORs for A-fib only, with relatively low signal strengths when compared to other drugs and outcomes. Although pharmacovigilance analyses cannot exclude rare adverse events or subgroup specific risks, for these two drugs, an absence of safety signal for MI, stroke, or CF, is reassuring, especially for patients at high risk for these events.^4,7–9,24–26^ Teriparatide similarly demonstrated a favorable cardiovascular reporting profile, consistent with prior clinical trial and observational evidence.^24–27^

This study has several strengths, including use of a large national pharmacovigilance database encompassing more than 30 million adverse event reports over a recent six-year period, which provides high sensitivity for detecting potential safety signals across multiple therapeutic classes and clinically relevant cardiovascular outcomes. Use of established disproportionality methods enables systematic signal detection and offers complementary insight beyond that available from randomised trials alone.^17–20,23^

Although FAERS data cannot establish causality, reporting disproportionality may reflect a range of factors beyond a true drug effect, including channelling of higher-risk patients to therapy, differences in baseline cardiovascular comorbidity, stimulated or preferential reporting following regulatory warnings, and exposure-related “newness” effects. Differences in the duration of market availability and cumulative exposure may also influence reporting patterns, as older agents such as alendronate have decades of widespread clinical use and substantially larger reporting volumes compared with newer therapies such as romosozumab or abaloparatide. FAERS does not allow assessment of temporal relationships or whether stroke events occurred among patients with A-fib, precluding mechanistic inference. FAERS data are subject to many forms of bias, such as indication bias, reporting bias, and stimulated reporting following regulatory actions or media attention. Exposure denominators, treatment duration, dose, and timing of events are not reliably captured, precluding estimation of incidence rates or causal inference. Reporting odds ratios reflect disproportionality rather than absolute risk and should be interpreted as hypothesis generating.^19,20^ The lack of detailed clinical covariates limits adjustment for confounding and necessitates cautious interpretation of observed associations.^17,18,23^ Interpretation of these findings is inherently complex, as osteoporosis therapies are commonly prescribed to older individuals with substantial comorbidity burden. Confounding by indication, baseline cardiovascular risk, and polypharmacy likely contribute to these observed patterns.

Despite these limitations, pharmacovigilance analyses remain a critical component of post-marketing drug safety evaluation. Importantly, the findings of this study should not be interpreted as discouraging treatment of osteoporosis. Osteoporosis is a serious and often undertreated condition associated with substantial morbidity, mortality, and loss of independence following fragility fractures. For most patients, the benefits of effective osteoporosis therapy in reducing fracture risk will outweigh potential cardiovascular concerns. Rather than avoiding treatment, these results prompt further analysis of risk, which can be used to inform medication selection for individuals at risk of MACE. Integrating cardiovascular risk stratification into clinical decision making may help optimise outcomes while ensuring that patients continue to receive therapies that meaningfully reduce fracture risk. Ultimately, addressing skeletal and cardiovascular health together through individualised care represents the most appropriate path forward in osteoporosis management.

## References

[1] Compston JE McClung MR Leslie WD. Osteoporosis. Lancet 2019 Jan 26;393(10169):364–76. 30696576 10.1016/S0140-6736(18)32112-3

[2] Humphrey MB Russell L Danila MI Fink HA Guyatt G Michaud K 2022 American College of Rheumatology guideline for the prevention and treatment of glucocorticoid-induced osteoporosis. Arthritis Care Res (Hoboken) 2023 Dec;75(12):2405–19. 37884467 10.1002/acr.25240

[3] Buttgereit F Palmowski A Bond M Adami G Dejaco C. Osteoporosis and fracture risk are multifactorial in patients with inflammatory rheumatic diseases. Nat Rev Rheumatol 2024 Jul;20(7):417–31. 38831028 10.1038/s41584-024-01120-w

[4] Seeto AH Tadrous M Gebre AK Lewis JR Fink HA Ebeling PR Evidence for the cardiovascular effects of osteoporosis treatments in randomized trials of post-menopausal women: A systematic review and Bayesian network meta-analysis. Bone 2023;167:116610. 36372197 10.1016/j.bone.2022.116610

[5] Saag KG Petersen J Brandi ML Karaplis AC Lorentzon M Thomas T Romosozumab or alendronate for fracture prevention in women with osteoporosis. N Engl J Med 2017;377:1417–27. 28892457 10.1056/NEJMoa1708322

[6] Cosman F Crittenden DB Adachi JD Binkley N Czerwinski E Ferrari S Romosozumab treatment in postmenopausal women with osteoporosis. N Engl J Med 2016;375:1532–43. 27641143 10.1056/NEJMoa1607948

[7] Cummings SR San Martin J McClung MR Siris ES Eastell R Reid IR Denosumab for prevention of fractures in postmenopausal women with osteoporosis. N Engl J Med 2009;361:756–65. 19671655 10.1056/NEJMoa0809493

[8] Gu HF Gu LJ Wu Y Zhao XH Zhang Q Xu ZR Yang YM. Efficacy and safety of denosumab in postmenopausal women with osteoporosis: A meta-analysis. Medicine (Baltimore) 2015 Nov;94(44):e1674. 26554766 10.1097/MD.0000000000001674 PMC4915867

[9] Seeto AH Abrahamsen B Ebeling PR Rodríguez AJ. Cardiovascular safety of denosumab across multiple indications: A systematic review and meta-analysis of randomized trials. J Bone Miner Res 2021;36:24–40. 32780899 10.1002/jbmr.4157

[10] Kim DH Rogers JR Fulchino LA Kim CA Solomon DH Kim SC. Bisphosphonates and risk of cardiovascular events: A meta-analysis. PLoS One 2015;10:e0122646. 25884398 10.1371/journal.pone.0122646 PMC4401508

[11] Liu S Tan Y Huang W Luo H Pan B Wu S. Cardiovascular safety of zoledronic acid in the treatment of primary osteoporosis: A meta-analysis and systematic review. Semin Arthritis Rheum 2024;64:152304. 37984227 10.1016/j.semarthrit.2023.152304

[12] Camm AJ. Review of the cardiovascular safety of zoledronic acid and other bisphosphonates for the treatment of osteoporosis. Clin Ther 2010 Mar;32(3):426–36. 20399982 10.1016/j.clinthera.2010.03.014

[13] Black DM Delmas PD Eastell R Reid IR Boonen S Cauley JA Once-yearly zoledronic acid for treatment of postmenopausal osteoporosis. N Engl J Med 2007;356:1809–22. 17476007 10.1056/NEJMoa067312

[14] D’Silva KM Cromer SJ Yu EW Fischer M Kim SC. Risk of incident atrial fibrillation with zoledronic acid versus denosumab: A propensity score-matched cohort study. J Bone Miner Res 2021 Jan;36(1):52–60. 33137852 10.1002/jbmr.4174 PMC7938865

[15] Bunch TJ Anderson JL May HT Muhlestein JB Horne BD Crandall BG Relation of bisphosphonate therapies and risk of developing atrial fibrillation. Am J Cardiol 2009;103:824–8. 19268739 10.1016/j.amjcard.2008.11.037

[16] Rubin KH Möller S Choudhury A Zorina O Kalsekar S Eriksen EF Cardiovascular and skeletal safety of zoledronic acid in osteoporosis: Observational, matched cohort study using Danish and Swedish health registries. Bone 2020;134:115296. 32097760 10.1016/j.bone.2020.115296

[17] Abrahamsen B Eiken P Brixen K. Atrial fibrillation in fracture patients treated with oral bisphosphonates. J Intern Med 2009 May;265(5):581–92. 19141097 10.1111/j.1365-2796.2008.02065.x

[18] Potter E Reyes M Naples J Dal Pan G. FDA Adverse Event Reporting System (FAERS) essentials: A guide to understanding, applying, and interpreting adverse event data reported to FAERS. Clin Pharmacol Ther 2025 Sep;118(3):567–82. 40384638 10.1002/cpt.3701 PMC12393772

[19] Evans SJ Waller PC Davis S. Use of proportional reporting ratios (PRRs) for signal generation from spontaneous adverse drug reaction reports. Pharmacoepidemiol Drug Saf 2001 Oct–Nov;10(6):483–6. 11828828 10.1002/pds.677

[20] Lu Z Suzuki A Wang D. Statistical methods for exploring spontaneous adverse event reporting databases for drug-host factor interactions. BMC Med Res Methodol 2023 Mar 27;23(1):71. 36973693 10.1186/s12874-023-01885-w PMC10041785

[21] Tobias JH. Sclerostin and cardiovascular disease. Curr Osteoporos Rep 2023 Oct;21(5):519–26. 37490188 10.1007/s11914-023-00810-w PMC10543142

[22] Rodríguez AJ Nerlekar N Ebeling PR. Cardiac adverse events in bisphosphonate and teriparatide users: An international pharmacovigilance study. Bone 2023 Mar;168:116647. 36543300 10.1016/j.bone.2022.116647

[23] Michel C Scosyrev E Petrin M Schmouder R. Can disproportionality analysis of post-marketing case reports be used for comparison of drug safety profiles? Clin Drug Investig 2017 May;37(5):415–22. 10.1007/s40261-017-0503-6 28224371

[24] Cosman F Peterson LR Towler DA Mitlak B Wang Y Cummings SR. Cardiovascular safety of abaloparatide in postmenopausal women with osteoporosis: Analysis from the ACTIVE phase 3 trial. J Clin Endocrinol Metab 2020 Nov 1;105(11):3384–95. 32658264 10.1210/clinem/dgaa450 PMC7500469

[25] Miller PD Hattersley G Riis BJ Williams GC Lau E Russo LA Effect of abaloparatide vs placebo on new vertebral fractures in postmenopausal women with osteoporosis: A randomized clinical trial. JAMA 2016;316:722–33. 27533157 10.1001/jama.2016.11136

[26] Cosman F Cooper C Wang Y Mitlak B Varughese S Williams SA. Comparative effectiveness and cardiovascular safety of abaloparatide and teriparatide in postmenopausal women new to anabolic therapy: A US administrative claims database study. Osteoporos Int 2022 Aug;33(8):1703–14. 35524068 10.1007/s00198-022-06413-y PMC9499892

[27] Cosman F Nieves JW Dempster DW. Treatment sequence matters: Anabolic and antiresorptive therapy for osteoporosis. J Bone Miner Res 2017 Feb;32(2):198–202. 27925287 10.1002/jbmr.3051

